# Biokinetic model of radioiodine I-131 in nine thyroid cancer patients subjected to in-vivo gamma camera scanning: A simplified five-compartmental model

**DOI:** 10.1371/journal.pone.0232480

**Published:** 2020-05-04

**Authors:** Chao-Chun Huang, Ya-Hui Lin, Samrit Kittipayak, Yi-Shi Hwua, Shan-Ying Wang, Lung-Kwang Pan

**Affiliations:** 1 Graduate Institute of Radiological Science, Central Taiwan University of Science and Technology, Taichung, Taiwan, ROC; 2 Department of Nuclear Medicine, Far Eastern Memorial Hospital, New Taipei City, Taiwan, ROC; 3 College of Nursing, Central Taiwan University of Science and Technology Takun, Taichung, Taiwan, ROC; 4 Department of Clinical Pharmacy, Taichung Armed Forces General Hospital, Taichung, Taiwan, ROC; 5 Department of Radiological Technology, Faculty of Medical Technology, Mahidol University, Bangkok, Thailand; Instituto de Investigacion Sanitaria de Santigao de Compostela, SPAIN

## Abstract

A five-compartmental biokinetic model of I-131 radioiodine based on in-vivo gamma camera scanning results was developed and successfully applied to nine thyroid cancer patients who were administered 1,110 MBq I-131 in capsules for the residual thyroid gland ablation. The I-131 solution activity among internal organs was analyzed via the revised biokinetic model of iodine recommended by the ICRP-30 and -56 reports. Accordingly, a five-compartmental (stomach, body fluid, thyroid, whole body, and excretion) model was established to simulate the metabolic mechanism of I-131 in thyroid cancer patients, whereas the respective four simultaneous differential equations were solved via a self-developed program run in MATLAB. This made it possible to provide a close correlation between MATLAB simulation results and empirical data. The latter data were collected through in-vivo gamma camera scans of nine patients obtained after 1, 4, 24, 48, 72, and 168 hours after radioactive I-131 administration. The average biological half-life values for the stomach, body fluid, thyroid, and whole body of thyroid cancer patients under study were 0.54±0.32, 12.6±1.8, 42.8±5.1, and 12.6±1.8 h, respectively. The corresponding branching ratios I_12_, I_23_, I_25_, I_34_, I_42_, and I_45_ as denoted in the biokinetic model of iodine were 1.0, 0.21±0.14, 0.79±0.14, 1.0, 0.1, and 0.9, respectively. The average values of the AT dimensionless index used to verify the agreement between empirical and numerical simulation results were 0.056±0.017, 0.017±0.014, 0.044±0.023, and 0.045±0.009 for the stomach, thyroid, body fluid + whole body, and total, respectively. The results obtained were considered quite instrumental in the elucidation of metabolic mechanisms in the human body, particularly in thyroid cancer patients.

## Introduction

This study is devoted to biokinetic model of radioiodine I-131 and its distribution in thyroid cancer patients using their in-vivo gamma camera scanning results. Thyroid cancer patients underwent surgical operation; then, their thyroid remnant tissues were ablated by high dose radiation from radioiodine I-131 [1–3]. Noteworthy is that as early as in 1978, guidance in a comprehensive analysis of the radioiodine activity in various human organs was proposed by the ICRP-56 report [4], which introduced the main criteria for the iodine biokinetic model elaboration and linked its results with related data on healthy persons. However, since that time, only scarce data on thyroid cancer were reported, which hindered the respective biokinetic model validation. The main stumbling block for more extensive research efforts seems to be the difficulty in quantifying the time-dependent concentration of radioiodine activity in each body compartment from the clinical viewpoint. Therefore, several feasible models defining five compartments (namely, stomach, body fluid, thyroid, whole body, and excretion) were introduced, which attempted to resolve this problem by solving a set of four time-dependent simultaneous differential equations. In particular, Chen *et al*. [5] analyzed five thyroid cancer patients via the gamma camera approach and presented a revised dataset for patients after surgical removal of the thyroid tissue. Hsu *et al*. [6] applied a similar technique to elaborate a biokinetic model of the gastrointestinal (GI) tract for 24 healthy volunteers. In contrast, Chiang *et al*. [7] surveyed five biokinetic models with a dynamic water phantom and gamma camera technique. The methodology of compiling the gamma camera technique with various theoretical biokinetic models became more straightforward and user-friendly after coupling the state-of-the-art gamma cameras with computed tomography (CT). This combination furnished more accurate identification of surveyed regions, i.e., borders of particular conventional compartments in patients’ bodies could be more readily distinguished.

In this paper, the biokinetic model simulation was combined with the gamma-scintigraphy survey of radioiodine-131 by the in-vivo gamma camera/4-slice CT technique for nine thyroid cancer patients who underwent the administration of radioiodine-131. These patients granted their written consent to participate in the biokinetic study of radioiodine and were surveyed by a gamma camera/4-slice CT during continuous seven days. The collected data were normalized and processed by a self-developed program run in MATLAB to pursue the optimal solution of four time-dependent simultaneous differential equations within the framework of the proposed five-compartmental biokinetic model. The results obtained were verified via the AT (i.e. agreement) dimensionless index to assess their computational accuracy and then compared to each other.

## Materials and methods

### Biokinetic model of radioiodine in the thyroid

**Fig 1:**
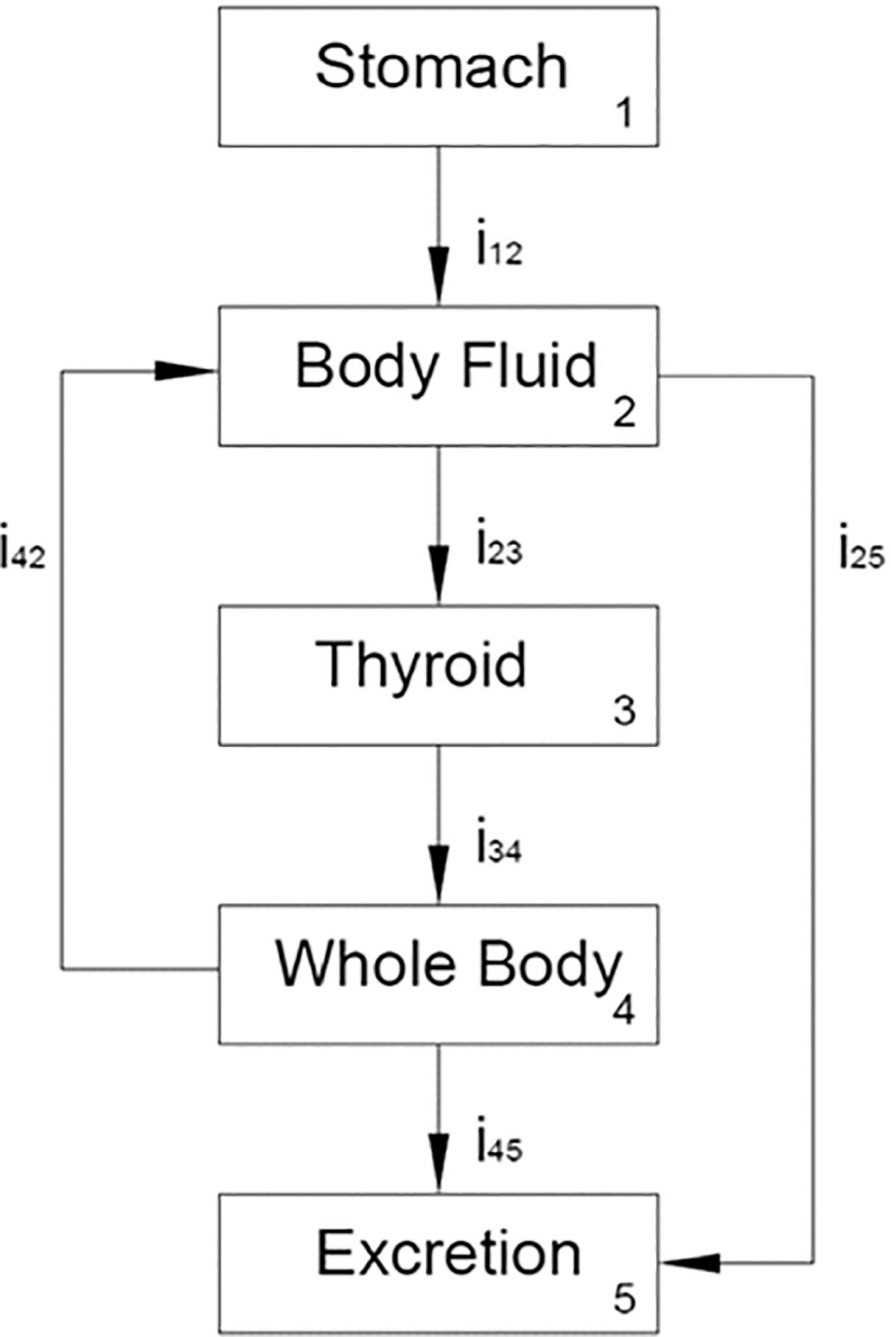
A typical human body can be divided into five major compartments in the biokinetic model of iodine: (1) stomach, (2) body fluid, (3) thyroid, (4) the whole body, and (5) excretion, according to the ICRP-30 report.

In compliance with the ICRP-56 report recommendations [4], a revised biokinetic model of radioiodine is proposed according to Eckeman’s original suggestion [8]. It implies a conventional subdivision of a typical human body into three major compartments, namely: (i) body fluid, (ii) thyroid, and (iii) whole body, whereas two compartments are newly defined in this study as (0) stomach and (iv) excretion. Since the radioactive I-131 is administered by giving radiopharmaceutical capsules to patients, the stomach is the initial organ, where the capsule is digested instantly. The last compartment is defined as excretion to hold the model into close range, and ensure the complete form of the respective numerical analysis [5–7]. This subdivision is schematically presented in Fig 1. To obtain the time-dependent correlation for each compartment, the following four simultaneous differential equations have to be solved.

$$\frac{dq_{1}}{dt}=-(\lambda_{P}+\lambda_{12})q_{1}$$ (1)

$$\frac{dq_{2}}{dt}=\lambda_{12}q_{1}-(\lambda_{P}+\lambda_{25}+\lambda_{23})q_{2}+\lambda_{42}q_{4}$$ (2)

$$\frac{dq_{3}}{dt}=\lambda_{23}q_{2}-(\lambda_{P}+\lambda_{34})q_{3}$$ (3)

$$\frac{dq_{4}}{dt}=\lambda_{34}q_{3}-(\lambda_{P}+\lambda_{42}+\lambda_{45})q_{4}$$ (4)

Here *q*_i_ is the time-dependent activity of the specific radionuclide; λ_ij_ is the biological decay constant (λ_ij_ = i_ij_·ln2/T_i(1/2)(bio)_); i_ij_ is the branching ratio of the infused radioactive solution from the i^th^ compartment to the j^th^ one; T_i(1/2)(bio)_ is the biological half-life of the i^th^ compartment, and λ_P_ is the physical decay constant of the specific radionuclide (namely, radioiodine). More detail can be found elsewhere [4, 7].

The biological half-life values of radioiodine recommended by Eckeman’s suggestion for the stomach, body fluid, thyroid, and the whole body are 0.696, 6, 1920, and 288 h, respectively. In addition, the branching ratios i_12_, i_23_, i_34_, i_45_, i_25_, and i_42_ were preset in Eckeman’s report as 1.0, 0.3, 1.0, 0.1, 0.7, and 0.9, respectively. Thus, the radioiodine activity for each compartment (cf. Fig 1) and the initial time correspond to the time when a dose of I-131 was administered to each patient. The system of (Eqs 1–4) was numerically solved via a self-developed program run in MATLAB [9]. However, the above parameters were validated only for healthy persons and are not applicable for thyroid cancer patients. Therefore, the biokinetic model of radioiodine for thyroid cancer patients had to be modified to represent their actual metabolic mechanism.

### Gamma camera SPECT/CT

This study used a gamma camera SPECT/CT (GE Healthcare Infinia Hawkeye 4 SPECT/CT) located at the Department of Nuclear Medicine, Far Eastern Memorial Hospital, New Taipei City, Taiwan, which involved an all-purpose, dual-detector, free-geometry integrated nuclear imaging camera. It was equipped with 59 circular photomultiplier tubes (either 53×76 mm^2^ or 6×38 mm^2^) connected to a 9.5 mm-thick NaI crystal inside each of two plates. The maximal field of view (FOV) of the gamma camera could be extended to 54×40 cm^2^ with an uncertainty of ± 0.5 mm. The auxiliary CT was preset to the spiral acquisition mode with amperages of 1.0, 1.5, 2.0, and 2.5 mA and tube voltages of 120 or 140 kV. The focal spot size could be switched from a small one (0.7×0.6 mm^2^) to a large one (0.9×0.9 mm^2^) and vice versa with the angular increment of 7^o^, according to the IEC 0 336 protocol [10]. During the scanning of each patient, two plates of NaI arrays of the gamma camera were positioned 5 cm above and 10 cm below the patient’s body, respectively. Ideally, the NaI detectors were supposed to capture ~70% of the emitted gamma-ray. The thyroid cancer patients under study were administered 1,110 MBq (30.0 mCi) ^I-131^ solution for the residual thyroid ablation. The I-131 solution was carrier-free with the radionuclide and radiochemical purity values exceeding 99.9 and 95.0%, respectively. All radiopharmaceutical capsules were fabricated by the *Global Medical Solutions*, *Ltd*. *(GMS*). The coefficient of variance (CoV) between solutions from the same fabricated batch was less than 1.0%, as confirmed by spot checks [11]. Thus, the position-sensitive gamma-ray emission from the I-131 dose administration to each subject under study could be robustly assessed and plotted for further analysis.

### Patients’ characteristics

Nine thyroid cancer patients (two males and seven females of 42~67 years of age) underwent post-thyroid cancer remnant ablation one month after the surgical operation. Each patient was subjected to a consecutive one-week whole-body scanning by gamma camera after the administration of I-131. Table 1 lists chacteristics of particular patients.

**Table 1:** Characteristics of nine patients with the papillary thyroid cancer diagnosis who underwent the whole-body scanning for further analysis of the iodine biokinetic model.

Patient No.	Gender	Age (yrs)	Weight, *W* (kg)	Height, *H* (cm)	Days after I-131 administration upon surgical operation
1	M	42	74	164	38
2	F	49	60	158	36
3	F	57	61	149	25
4	F	57	54	145	35
5	F	56	55	165	33
6	F	67	65	156	32
7	F	48	50	155	29
8	F	61	58	151	35
9	M	51	101	168	36
Average		52.8±8.4	62.5±15.5	156.1±7.6	33.2±4.1

### Whole-body scanning of patients

**Fig 2:**
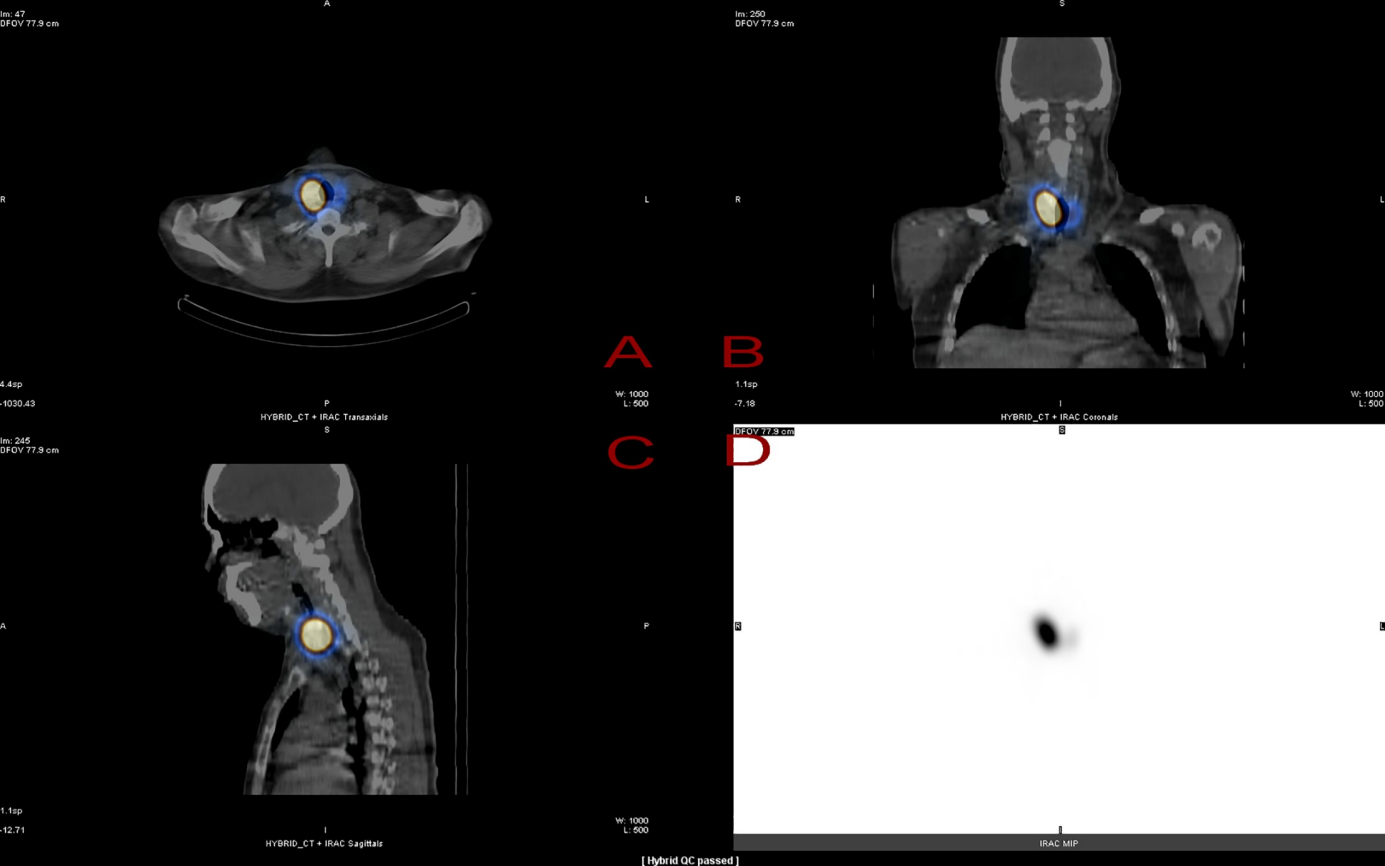
The SPECT/CT fusion plot of the residual thyroid gland for patient No. 1 is displayed as (A) axial view, (B) coronal view, (C) sagittal view of SPECT/CT fusion plot, and (D) maximum intensity projection (MIP), corresponding to the iterative reconstruction with the attenuation correction (IRAC) of the residual thyroid gland.

Each patient was given 1,110 MBq I-131 solution via a radiopharmaceutical capsule one hour before the in-vivo gamma camera scanning, and the preset schedule was at 1, 4, 24, 48, 72, and 168 elapsed hours for gaining six data groups for each patient. The preset protocol of gamma camera for data collection was ±10% peak width of the I-131 main 364 keV peak, high-energy general-purpose (HEGP) collimator, FOV of 40×54 cm^2^, and 256×1024 matrix size. Each patient underwent 20~22 min scanning with a 10 cm/min scan speed. The survey of this study was approved by the IRB Committee of the Far Eastern Memorial Hospital with the credential No. FEMH 106132-F. Besides, a SPECT/CT fusion plot was mandatory before the gamma scanning of each patient, to ensure the exact position of the marked ROI (region of interest) of the residual thyroid gland for further analysis. The scan protocol of 4-sliced CT was preset as 140 kV, 2.5 mA, 23 sec, spin width of 10 mm, spiral speed of 0.435 mm/s, and matrix size of 512×512. The original fusion plot of the residual thyroid gland for patient No. 1 is presented in Fig 2, which contains (A) axial view, (B) coronal view, (C) sagittal view of SPECT/CT fusion plot, and (D) maximum intensity projection (MIP), corresponding to the iterative reconstruction with the attenuation correction (IRAC) of the residual thyroid gland.

### AT examination

The scanned data on various ROIs from the gamma camera were collected and converted to count×pixel^-1^×s^-1^. Then, the maximal ROI_ST_ (the first acquired data from stomach after one hour upon the I-131 dose administration) was set as standard 1.0 to normalize the total dataset for each patient. Further, all the empirical data after normalization were assigned as the ideal objective expectation of dataset for optimization of the program prediction to obtain the parameter. An agreement value (AT) earlier adopted by the authors in [5, 7, 12] was used to determine the similarity between the empirical data and respective optimal predictions made via the self-developed program run in MATLAB. This AT-based approach, which was an extension from the definition of conventional RMS (root mean square) method, was beneficial in evaluating the fluctuation among large datasets because a quantified AT value was focused not only on the difference between particular empirical and theoretical results but also on the integration of all data in the same group. Thus, in the field of precision machining, a derived RMS was used to assess the surface roughness of large-area workpieces [13]. The agreement value AT was defined in [5, 7, 12] as follows:

$${AT}_{i}=\sqrt{\frac{\sum_{i=1}^{n}{\left[Y_{i}(nor.)-Y_{i}(MATLAB)\right]}^{2}}{N}}$$ (5)

where Y_i_ (nor.) and Y_i_ (MATLAB) are the normalized intensities from each ROI determined from the *n*-th set of empirically obtained data, which were computed using the self-developed program run in MATLAB. The value of *N* = 6 was adopted, insofar as the empirical data were collected in six independent scan groups. A zero value of AT of implies a perfect agreement between the theoretical and empirical results, while small AT always indicates an excellent consistency between them, meaning that the empirical data fluctuation from the predicted values is not large.

## Results

### Biokinetic model

**Fig 3:**
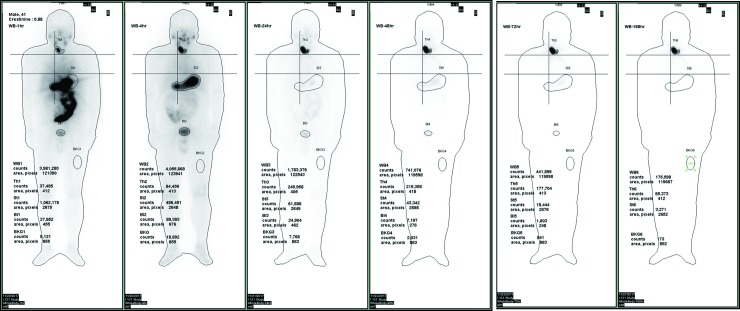
The original plot from the gamma camera of patient No. 1. **The six plots were consecutively downloaded from the facility after the gamma scanning after 1, 4, 24, 48, 72, and 168 elapsed hours, respectively.** The marked ROIs were assigned as St (stomach), WB (body fluid + whole body), Th (thyroid), Bl (bladder), and BKG (background) to derive the count×pixel^-1^×s^-1^ for further analysis.

**Fig 4:**
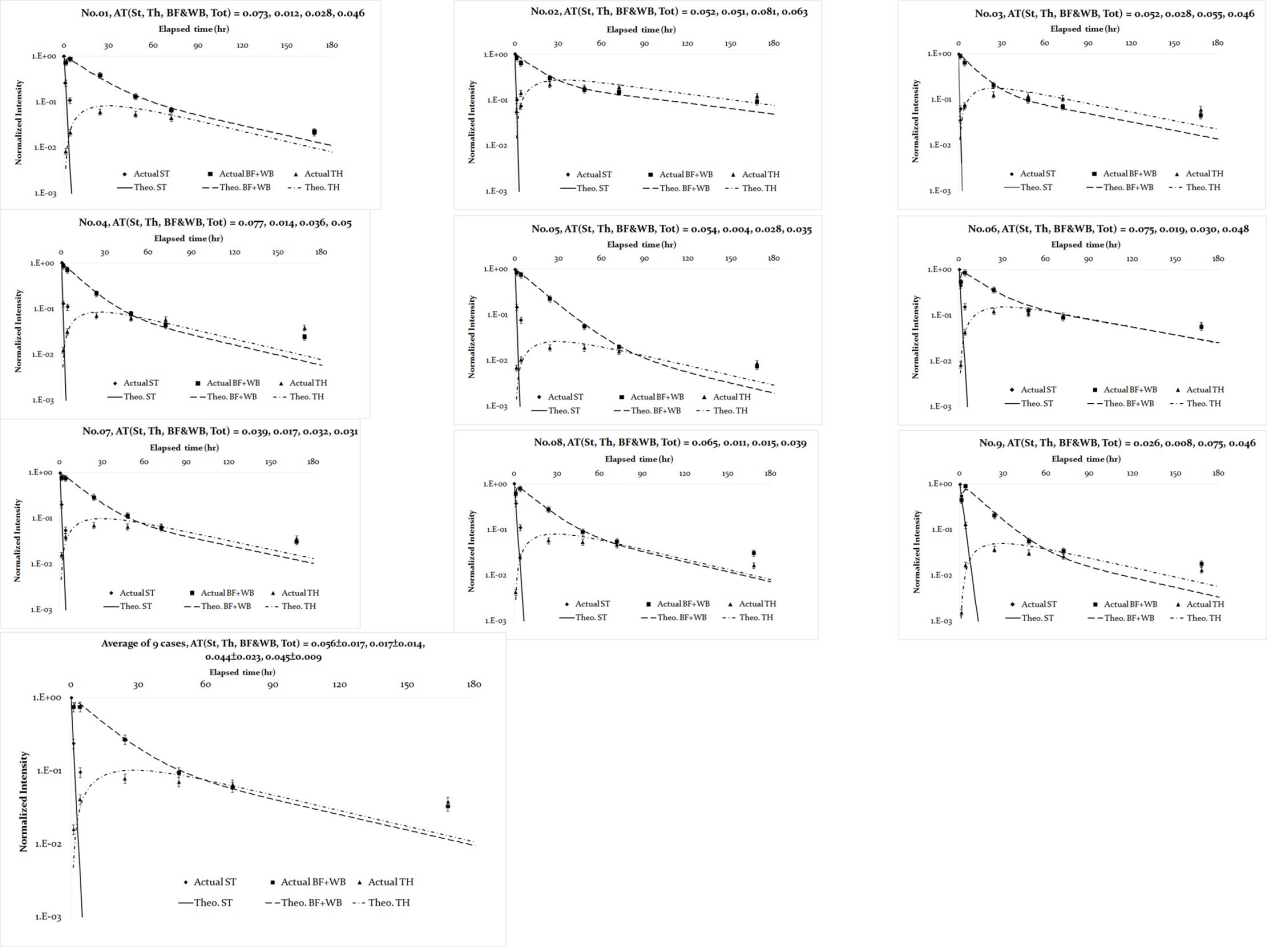
The normalized empirical data for patients and theoretical estimates via the MATLAB program. The former ones are illustrated by dots with error bars, and the optimal estimates are plotted with three continuous lines.

Fig 3 shows six original plots constructed from the gamma camera scans of patient No. 1 taken after 1, 4, 24, 48, 72, and 168 elapsed hours, respectively. The marked ROIs were assigned as St (stomach), WB (body fluid (BF) +whole body (WB)), Th (thyroid), Bl (bladder), and BKG (background) to derive the value of count×pixel^-1^×s^-1^ for further analysis. Besides, the BKG values were subtracted from all net count rates at various ROIs, in order to eliminate the over-counting in the analysis (cf. Fig 3). All these were split into counts and pixels, with the following normalization by the respective maximal value obtained for the stomach after one hour upon the dose administration. The above normalization was mandatory for integrating the empirical data with the results calculated via the self-developed program run in MATLAB. Table 2 summarizes the derived biological half-lives and variables for simultaneous time-dependent differential equations (cf. (Eqs 1–4)), as adopted in this study. The calculation results gave theoretical estimations of the time-dependent quantity of I-131 in various compartments for nine patients and were averaged, according to the MATLAB calculation. Additionally, the respective parameters for a standard healthy adult male, as recommended by Eckeman [8], are also given for comparison. Fig 4 integrates the normalized empirical data for patients (dots with error bars) and theoretical estimates via MATLAB (continuous, dashed, and dot-and-dash lines for St, BF+WB, and Th, respectively). The lines correspond to stable and converged solutions derived via multiple iterations of the Gaussian elimination, according to the inverse problem algorithm. Thus, the optimal solution provided a better fit of four simultaneous time-dependent differential (Eqs 1–4) than any individual one.

**Table 2:** The derived biological half-life values and variables for simultaneous time-dependent differential equations (cf. Eqs 1–4) as adopted in this study. The calculation results are theoretical estimations of the time-dependent quantity of I-131 in various compartments for nine patients, according to the MATLAB calculation.

Patient No.	T_1/2(bio)_ [h]			Branching ratio					
	St	BF&WB	Th	I_12_	I_23_	I_25_	I_34_	I_42_	I_45_
**Ref. [8]**	**0.696**	**6**_**&**_**288**	**1920**	**1.0**	**0.30**	**0.70**	**1.0**	**0.9**	**0.1**
1	0.50	15	35	1.0	0.18	0.82	1.0	0.9	0.1
2	0.30	14	50	1.0	0.50	0.50	1.0	0.9	0.1
3	0.30	10	40	1.0	0.30	0.70	1.0	0.9	0.1
4	0.33	11	40	1.0	0.15	0.85	1.0	0.9	0.1
5	0.35	12	50	1.0	0.04	0.96	1.0	0.9	0.1
6	0.60	15	40	1.0	0.30	0.70	1.0	0.9	0.1
7	0.45	13	45	1.0	0.18	0.82	1.0	0.9	0.1
8	0.70	12.5	40	1.0	0.15	0.85	1.0	0.9	0.1
9	1.30	11	45	1.0	0.08	0.92	1.0	0.9	0.1
**Average**	**0.54±0.32**	**12.6±1.8**	**42.8±5.1**	**1.0**	**0.21±0.14**	**0.79±0.14**	**1.0**	**0.9**	**0.1**

### AT analysis

Fig 4 also displays the AT values of the derived compartments for each of nine patients, which indicate the curve-fitting agreement between theoretical estimations and empirical measurements for each specific compartment of patient’s body. For all patients under study, their AT for ST, Th, and BF+WB fluctuated in the range of 0.002~0.08 Besides, the total AT was calculated as the mean square root of the three derived ATs (AT_tot_ = $\sqrt{({AT}_{St}^{2}+{AT}_{Th}^{2}+{AT}_{BF+WB}^{2})/3}$ = 0.045±0.009). Such reasonable confidences prove that a complicated correlation (coupling) occurred between various compartments, which contributed to the enhanced integration of empirical survey and theoretical estimation results.

## Discussion

### Biokinetic model of radioiodine for thyroid cancer patients

The results obtained via the biokinetic model of radioiodine for thyroid cancer patients was found to strongly deviate from those recommended by Eckeman’s suggestion for healthy male adults (HMA). The comparative analysis of data listed in Table 2 revealed that the biological half-lives of the stomach for thyroid cancer patients and HMA were quite close (0.54±0.32 versus 0.696 h, respectively), while those for body fluid, whole body, and thyroid exhibited a large deviation. While Eckeman’s report implied T_1/2_(BF) = 6h, T_1/2_(thyroid) = 1920h, and T_1/2_(WB) = 288h, this study predicted T_1/2_(BF+WB) = 12.6±1.8h and T_1/2_(thyroid) = 42.8±5.1h (cf. Tab. 2). Thus, the activity of I-131 in two compartments (BF+WB) exhibited a rapid degradation at the initial stage after the I-131 administration because of a short effective half-life of the (BF+WB) compartment and then rose gradually, due to a long effective half-life of the thyroid compartment. This pattern is similar to the transient equilibrium in the radioactive chain decay [14]. In addition, the average biological half-life of either BF or WB was 12.6±1.8h in this study. The precise decay trend among compartments of each patient reflects an accurate prediction by the self-developed program run in MATLAB and shows its potential application in surveying other similar syndromes with a slight refinement of the program.

### Comparison with other available biokinetic models

Table 3 summarizes the correlated biological half-lives of either whole body or residual thyroid gland for thyroid cancer patients from other studies. The obtained data were collected from practical surveys or derived by the theoretical estimation. As clearly depicted, the biological half-lives of the whole body fluctuated in the range of 6~30h, except for one patient with renal failure syndrome listed in Willegaignon’s clinical case report, where this value was equal to 45.5h [15]. Noteworthy is that Chen *et al*. adopted a similar method to survey the biokinetic model of five thyroid cancer patients and reported that the biological half-life of either whole body or thyroid was 12.5±5.5 or 15.8±24.0h [5]; whereas Guiu-Souto *et al*. adopted the initial values of 6 and 480h, respectively, in their iterative fitting algorithm based on the theoretical simulation of HMA using a similar assumption in the ICRP-53 report [19]. However, due to the normal metabolic mechanism in the HMA thyroid gland, the fact that the latter holds the radioiodine for a relatively long time seems to be quite realistic. Although the effective biological half-life of I-131 in the thyroid is still 135h (1/135 = 1/192 +1/450), the derived value of 450h is just a simulated result with insignificant meaning in the practical survey. Most analytical assessments listed in Table 3 were made based on the exponential decay of two compartments and defined as instant or slow decay terms for the numerical analysis [15–18, 20]. The most intricate model of Guiu-Souto *et al*. [19] included ten compartments and assigned two feedback paths (from body fluid to stomach and liver to body fluid), whereas others either had no assigned feedback path or defined only one feedback path. Meanwhile, a short biological half-life of the whole body reported by Guiu-Souto *et al*. [19] can be attributed to a non-equilibrium status with the daughter compartment (thyroid) in their unique biokinetic model. Thus, the daughter compartment (thyroid) dominated the whole decay characteristic. The latter one (from the whole body to body fluid, cf. Fig 1) was adopted in this study, as well. The assigned feedback path in the biokinetic model may complicate the analytical simulation in compiling with the practical data from gamma camera scanning. However, the derived results comply with the true metabolic mechanism, according to Eckeman’s report. In addition, ignoring the feedback path in a biokinetic model may also misjudge the contribution from either body fluid or whole body for thyroid cancer patients, since most of them have only the residual thyroid gland, which performance strongly differs from fully-functional thyroid glands of HMA. However, incorporation of even a single feedback path into a biokinetic model definitely complicates the analysis and integration of the empirical data.

**Table 3:** The derived biological half-lives of either whole-body or residual thyroid gland, as well as the internal doses for thyroid cancer patients from other studies.

Ref.	Compartment (organs)	T_1/2(bio)_ [h]	Subject
[5]	Thyroid	15.8±24.0	Five thyroid cancer patients
	Whole-body	12.5±5.5	
[15]	Whole-body	45.5	One thyroid cancer patient with renal failure
[16]	Whole-body	19.4 (initial) 360 (after two weeks)	Ten female thyroid cancer patients
[17]	Thyroid	63.2±44.9	36 thyroid cancer patients
	Whole-body	13.7±2.5	
[18]	Whole-body	30±17	12 female thyroid cancer patients
[19]	Thyroid	480	The initial values of the iterative fitting algorithm as adopted in the theoretical simulation according to the ICRP-53
	Whole-body	6	
[20]	Whole-body	25.3	118 thyroid cancer patients
	Whole-body	13.4	Eight metastatic lymph nodes’ patients
This study	Thyroid	38.7±13.8	Nine thyroid cancer patients
	Whole-body	12.4±1.9	

## Conclusion

The biokinetic model of I-131 radioiodine for nine thyroid cancer patients who underwent post-thyroid cancer remnant ablation was developed and applied, according to in-vivo gamma camera scanning results. Nine thyroid cancer patients were administered 1,110 MBq I-131 for the residual thyroid gland ablation. The undertaken radioiodine concentration distributed among the internal organs was analyzed according to the biokinetic model that followed the recommendation of Eckeman. In doing so, a five-compartmental-model was established to simulate the metabolic mechanism of thyroid cancer patients, and a self-developed program run in MATLAB was elaborated to solve four simultaneous differential equations. The latter yielded the optimal solution, which complied with the empirical findings made via the gamma camera scanning and exhibited a high reproducibility.

## Supporting information

S1 Data (XLSX)
